# A Cautionary Tale: Endogenous Biotinylated Proteins and Exogenously-Introduced Protein A Cause Antibody-Independent Artefacts in Western Blot Studies of Brain-Derived Proteins

**DOI:** 10.1186/s12575-019-0095-z

**Published:** 2019-04-18

**Authors:** Marianne K. O. Grant, Samantha L. Shapiro, Karen H. Ashe, Peng Liu, Kathleen R. Zahs

**Affiliations:** 1 Departments of Neurology, University of Minnesota, Minneapolis, MN 55455 USA; 2 Departments of Neuroscience, University of Minnesota, Minneapolis, MN 55455 USA; 3 N. Bud Grossman Center for Memory Research and Care, University of Minnesota, Minneapolis, MN 55455 USA; 4 GRECC, VA Medical Center, Minneapolis, MN 55417 USA; 5 Present address: Department of Experimental and Clinical Pharmacology, University of Minnesota, Minneapolis, MN 55455 USA; 6 Present address: University of Wisconsin – Madison, Madison, WI 53705 USA

**Keywords:** Western blotting, Biotin, Protein A, Artefact

## Abstract

Antibodies are commonly used to detect or isolate proteins from biological samples. Much attention has been paid to the potential for poorly-characterized antibodies to lead to misleading results, but *antibody-independent* artefacts may also occur. Here, we recount two examples of antibody-independent artefacts that have confounded the interpretation of results in our search for molecular entities associated with memory loss in Alzheimer’s disease (AD). First, when using biotin-avidin systems for antibody detection, endogenous biotinylated proteins created spurious bands in Western blots of brain lysates from AD patients and transgenic mouse models of AD. These artefactual bands occurred in a transgene- and strain-dependent manner. A second, unexpected artefact occurred when Protein A-conjugated Sepharose beads were used to deplete lysates of endogenous immunoglobulins prior to immunopurification of target proteins. In these assays, Protein A shed from the beads, then bound to (and was eluted from) an immunoaffinity matrix designed to capture AD-related proteins. The Protein A then bound detection antibodies when the immunoaffinity eluates were analyzed by Western blot. Both of these artefacts–the endogenous biotinylated proteins and the Protein A artefact–can be monitored by including an “irrelevant” antibody as an experimental control (e.g., running a parallel protocol in which the antibody directed against the target of interest is replaced by a non-specific antibody).

**Electronic supplementary material:**

The online version of this article (10.1186/s12575-019-0095-z) contains supplementary material, which is available to authorized users.

## Background

In 2011–2012, the scientific community was shaken by the publication of two articles from scientists at Bayer Health [1] and Amgen [2], who reported that their attempts to confirm published preclinical findings were successful in only 20–25% (of 67) and 11% (of 53) of projects, respectively. These papers ignited discussion of a “reproducibility crisis” in science–a discussion that continues to this day in both the scientific and popular press. A 2016 survey conducted by the journal *Nature* found that 90% of the > 1500 researchers surveyed agreed that there *is* a reproducibility crisis, with > 70% reporting that, at one time or other, they were unable to reproduce others’ experimental results, and > 50% reporting that they were unable to reproduce *their own* results [3]. Several factors have been proposed to contribute to this crisis, including funding and publishing incentives, misuse and/or misunderstanding of statistics, and poor experimental design [4–8]. Unvalidated reagents–particularly antibodies–have received a great deal of attention in the scientific press [9, 10], although, interestingly, reagent quality was not thought to be a major contributor to the lack of reproducibility among respondents in the *Nature* survey. Greater than 90% of *Nature* respondents believed that “more robust experimental design” would lead to greater reproducibility.

Immunocapture and immunoblotting are commonly used to isolate, identify and quantify proteins in biological sample (a PubMed search for “Western blot” conducted on March 14, 2019 returned 310,301 hits). We believe that there has been an unfortunate drift away from robust design in immunoblotting experiments–at least in our field of Alzheimer’s disease (AD) research, it does not appear that the use of methodological controls is routine when conducting immunoblotting experiments, despite the calls for more rigorous experimental design. We recently surveyed more than 500 studies (Additional file 1: Figure S1) that employed immunoblotting or immunocapture (immunoprecipitation or immunoaffinity purification) techniques, and found that only 22 of 503 (4.4%) studies reported a methodological control for immunoblotting (e.g., the use of an irrelevant antibody as the primary detection antibody or antigen pre-absorption); methodological controls (e.g., non-specific immunoglobulin for capture) in immunocapture experiments were more common (36 of 81, or 44%), but not universal. The lack of methodological controls can lead to the misinterpretation of experimental results, even when appropriate “sample controls” (e.g., disease vs. healthy control, transgenic vs. non-transgenic animal, drug-treated vs. vehicle-treated animal) are employed.

Here, we recount two examples of artefacts that we have encountered while conducting pilot studies or attempting to reproduce published results. While the first example might have been anticipated based on reports in the literature [11–13], the second was unexpected. In the first case, spurious bands were seen in Western blots when using biotin-avidin systems for antibody detection, due to the presence of endogenous biotinylated proteins. These bands were present in samples from transgenic animals but not non-transgenic controls, and could have easily been misinterpreted in the absence of methodological controls. In the second example, Western blots were used to identify species captured by immunoaffinity purification–here an artefactual band was caused by the introduction of exogenous proteins that were retained during immunoaffinity purification steps and that non-specifically bound (detection) antibodies. In this case, the inclusion of a sample control was sufficient to reveal the artefact, and methodological controls confirmed it.

While these specific illustrations are derived from our studies attempting to identify molecules associated with memory loss in AD, there is the potential for generating comparable artefacts whenever similar techniques are used. It is our hope that these illustrations will serve as a warning to other researchers conducting immunoblotting experiments and will encourage them to incorporate controls that they might have otherwise overlooked.

## Methods

### Human Brain Extracts

Human brain samples were obtained from the Minneapolis Veterans Administration Medical Center. Tissue blocks containing the temporal pole were subjected to a sequential extraction procedure [14]. Tissue was first gently dissociated in buffer 1 (50 mM tris(hydroxymethyl)aminomethane-hydrochloric acid (Tris–HCl), pH 7.6, 0.01% (volume/volume (v/v)) Nonidet P-40 (NP-40), 150 mM NaCl, 2 mM ethylenediaminetetraacetic acid (EDTA), 0.1% (weight/volume (w/v)) sodium dodecyl sulfate (SDS); with protease and phosphatase inhibitors (0.1 mM phenylmethylsulfonyl fluoride (PMSF); 0.2 mM 1,10-phenanthroline monohydrate (1,10-PTH); protease inhibitor cocktail (Cat # P8340, Sigma-Aldrich, St. Louis, MO (currently MilliporeSigma, Burlington, MA)); and phosphatase inhibitor cocktails (Cat #s P2850 and P5726, Sigma-Aldrich)). The tissue suspension was then centrifuged at 800×*g* for 10 min at 4 °C. The resulting pellet was suspended in buffer 2 (50 mM Tris–HCl, pH 7.4, 150 mM NaCl, 0.1% (v/v) Triton X-100, with protease and phosphatase inhibitors), followed by centrifugation (16,100×*g*, 90 min, 4 °C). This pellet was suspended in buffer 3 (50 mM Tris–HCl, pH 7.4, 150 mM NaCl, 0.5% (v/v) Triton X-100, 1 mM EDTA, 3% (w/v) SDS, 1% (w/v) deoxycholate, with protease and phosphatase inhibitors), nutated for 15 min (4 °C) to further cell lysis, then centrifuged (16,100×*g*, 90 min, 4 °C). The resulting supernatant was depleted of endogenous immunoglobulins using Protein-G Fast Flow Sepharose beads (Cat # 17061801, GE Healthcare Life Sciences, Marlborough, MA; 50 μL 1:1 slurry per 0.75–1.0 mL extract, 1 h, 4 °C), then stored at − 20 °C. Protein concentrations were determined immediately before immunoprecipitation/Western blotting, using a bicinchoninic acid (BCA) protein assay kit (Cat # 23225, Thermo Scientific, Rockford, IL).

### Mouse Brain Extracts

Lysates were prepared from the brains of Tg6209 mice [15] and age-matched, non-transgenic animals, using either the protocol described above (three-step extraction protocol) or an alternative (one-step) protocol. For one-step extraction, each hemi-forebrain was mechanically dissociated in 1 mL buffer 3. The resulting homogenate was nutated for 1 h (4 °C), and then centrifuged (16,100×*g*, 90 min, 4 °C). Supernatants were then frozen for at least 24 h, then thawed and centrifuged (16,100×*g*, 90 min, 4 °C). Supernatants from this centrifugation step were depleted of endogenous immunoglobulins using Protein-G Fast Flow Sepharose beads, as described above. Protein concentrations were determined by a BCA protein assay kit (Thermo Scientific).

Additional lysates were prepared from the brains of hAPP-J20 mice [16] and age-matched, non-transgenic animals, using the three-step protocol described above. The brain extracts using buffer 1 were used for immunoaffinity purification. Lysates were depleted of endogenous immunoglobulins using Fast Flow Sepharose beads conjugated to native Protein A (Cat # 17528006, GE Healthcare Life Sciences), recombinant Protein A (Cat # 17127901, GE Healthcare Life Sciences), or Protein G.

### Immunoprecipitation (IP)

Amyloid precursor protein (APP) metabolites were immunoprecipitated from brain lysates using monoclonal antibody 6E10 (Cat # SIG-39320, Covance, Princeton, NJ (currently Cat # 803003, BioLegend, San Diego, CA)), which recognizes amino acids 6–10 of amyloid-β (Aβ). Frozen lysates were thawed on ice, then centrifuged at 9600×*g* for 20 min, 4 °C to remove any cryoprecipitates. Samples containing 50–100 μg protein were brought to a volume of 500 μL through the addition of immunoprecipitation dilution buffer (IPDB; 50 mM Tris-HCl, 150 mM NaCl, pH 7.4) containing protease and phosphatase inhibitors (0.1 mM PMSF; 0.2 mM 1,10-PTH; protease inhibitor cocktail, and phosphatase inhibitor cocktails, all from Sigma-Aldrich). Samples were then incubated overnight at 4 °C with 6E10 capture antibody (5 μg/reaction) and Protein G-coated magnetic beads (Dynabeads, Cat # 10004D, Life Technologies, Grand Island, NY (currently Cat # 10004D, Thermo Fisher Scientific, Waltham, MA); 50 μL slurry per reaction). Buffer was then removed, and beads were washed for 20 min at 4 °C with immunoprecipitation buffer A (IP buffer A; 50 mM Tris-HCl, 300 mM NaCl, 1 mM EDTA, 0.1% (v/v) Triton X-100, pH 7.4), and then for 20 min at 4 °C with immunoprecipitation buffer B (IP buffer B; 50 mM Tris-HCl, 150 mM NaCl, 1 mM EDTA, 0.1% (v/v) Triton X-100, pH 7.4). Proteins were eluted by boiling in 30 μL SDS- polyacrylamide gel electrophoresis (PAGE) loading buffer (450 mM Tris-HCl, pH 8.0, 24% (v/v) glycerol, 8% (w/v) SDS, 0.1% (w/v) bromophenol blue, 0.1% (v/v) Phenol Red; 5% (v/v) β-mercaptoethanol).

### Preparation of Immunoaffinity Matrices

Monoclonal antibodies 6E10 (200 μg) or a combination of 13.1.1 and 2.1.3 (anti-Aβ_40_ and anti-Aβ_42_, respectively, 100 μg each, gift of Dr. Pritam Das, Mayo Clinic, Jacksonville, FL) were covalently linked to Protein-G coupled magnetic beads (500 μL bead slurry, Protein G Mag Sepharose, Cat # 28951379, GE Healthcare Life Sciences), according to the following protocol. Beads were washed three times with IPDB containing protease and phosphatase inhibitors (1 mL buffer/500 μL bead-slurry equivalent), then re-suspended in 1 mL IPDB with protease inhibitors added (0.1 mM PMSF, 0.2 mM 1,10-PTH, and 1× protease inhibitor cocktail, Sigma-Aldrich). Antibodies were then added, and the mixture was incubated overnight at 4 °C, with mixing. Buffer with unbound antibody was then removed, and beads were washed one time with 1 mL IPDB, and one time with 1 mL triethanolamine (TEA, 200 mM, pH 9.0). Beads were then incubated for 15 min, room temperature, with mixing, in 1 mL 200 mM TEA with 50 mM dimethyl pimelimidate dihydrochloride, in order to crosslink the antibodies to the beads. Following crosslinking, beads were washed once with 1 mL 200 mM TEA, pH 9.0, and then the reaction was quenched by incubating the beads in 1 mL 10% (v/v) ethanolamine, for 15 min at room temperature, with mixing. The quenching solution was then removed, and the beads were washed once with 100 mM glycine-HCl, pH 2.8 with 2 M urea, and then three times with IPDB (1 mL solution per wash). Beads were stored in 12 mL IP buffer B with the protease inhibitors listed above, at 4 °C.

### Immunoaffinity Purification

Frozen mouse brain lysates were thawed on ice, and then centrifuged at 9600×*g* for 10 min, 4 °C to remove any cryoprecipitates. Lysate (containing 1.5–2 mg protein) was added to the anti-Aβ_40/42_ immunoaffinity matrix (with fresh protease inhibitors added), and incubated overnight at 4 °C, with mixing. The buffer was then removed, and the beads were washed with 12 mL IP buffer B with 1% (v/v) Triton X-100. Captured proteins were then eluted using 333 μL elution buffer (33 mM glycine-HCl, 1% (w/v) Octyl *β*-D-1-thioglucopyranoside (Cat # O6004, Sigma-Aldrich), pH 2.8). The elution step was repeated two more times.

Additionally, some lysates were subjected to a sequential immunopurification protocol in which lysate was first applied to a 6E10 immunoaffinity matrix, and then the eluate from this matrix was applied to the anti-Aβ_40/42_ matrix. Lysate (containing 1.5–2 mg protein) was added to the 6E10 immunoaffinity matrix (with fresh protease inhibitors added), and incubation, wash, and elutions then proceeded as described above. The three eluates from the 6E10 matrix were pooled, and protease inhibitors were added. Pooled eluates were concentrated approximately 2X in a Vacu-fuge, 35 μL was removed for Western blotting, and the remaining eluate was applied to the anti-Aβ_40/42_ immunoaffinity matrix. Incubation, wash, and elutions then proceeded as described above.

### Western Blotting (WB)

Proteins were denatured by boiling for 5 min in SDS-PAGE loading buffer (note that bromophenol blue was omitted from the loading buffer for blots visualized using the Li-Cor system), then size-fractionated on 10–20% Tris-Tricine precast gels (Cat # 345–0067, Bio-Rad, Hercules, CA) and electrophoretically transferred onto 0.2-μm nitrocellulose membranes at 0.4 A for 3 h at 4 °C. Membranes were boiled twice in phosphate-buffered saline (Cat # P4417-100TAB, Sigma-Aldrich)–for 25 s and 15 s, with a 4-min interval between periods of boiling, blocked with blocking buffer (5% (w/v) bovine serum albumin (Cat # A3803-100G, Sigma-Aldrich) in Tris-buffered saline (TBS; 10 mM Tris-HCl, pH 7.4, 200 mM NaCl) with 0.1% (v/v) Tween 20 (TBS-T)) for 1 h at room temperature, and then incubated overnight at 4 °C with one of the detection antibodies listed in Table 1 (all antibodies were diluted in blocking buffer). Following this overnight incubation, membranes were washed 5 times in TBS-T (5 min per wash) at room temperature and further processed according to the detection antibody used.

**Table 1:** Antibodies

Antibody	Format	Isotype	Vendor	Product Number
Anti-Aβ_40_	Affinity-purified monoclonal	Mouse IgG1, κ	Gift of Dr. Pritam Das	13.1.1
Anti-Aβ_42_	Affinity-purified monoclonal	Mouse IgG1, κ	Gift of Dr. Pritam Das	2.1.3
Anti-APP(C-terminal)	Affinity-purified monoclonal	Rabbit IgG	Invitrogen^a^	36–6900
1G6-biotin	Affinity-purified monoclonal	Mouse IgG2b	Covance^b^	SIG-39181
4G8	Affinity-purified monoclonal	Mouse IgG2b, κ	Covance^b^	SIG-39220
6E10	Affinity-purified monoclonal	Mouse IgG1	Covance^b^	SIG-39320
6E10-biotin	Affinity-purified monoclonal	Mouse IgG1	Covance^b^	SIG-39340
82E1-biotin	Affinity-purified monoclonal	Mouse IgG1	IBL America (Minneapolis, MN)	10,326
A11	Rabbit serum	NA^c^	Gift of Dr. Charles Glabe	
22C11	Affinity-purified monoclonal	Mouse IgG1	EMD Millipore^d^	MAB348
Anti-Protein A-biotin	Affinity-purified monoclonal	Mouse IgG1	Sigma-Aldrich^d^	B-3150
Anti-α-tubulin	Affinity-purified monoclonal	Mouse IgG1	Sigma-Aldrich^d^	T-5168

^a^now Thermo Fisher Scientific (Waltham, MA); ^b^now Biolegend (San Diego, CA); ^c^NA = not applicable; ^d^now MilliporeSigma (Burlington, MA)

When biotin-conjugated detection antibodies were used, blots were incubated for 5–10 min at room temperature with Neutravidin-horseradish peroxidase (HRP) (1:5000 in TBS-T, Cat # A2664, Invitrogen (currently Thermo Fisher Scientific)) or Streptavidin-IRDye 800CW (1:5000; Cat # P/N 926–32,230, Li-Cor, Lincoln, NE), then washed 5 X 5 min in TBS-T. Blots exposed to Neutravidin-HRP were then developed using a chemiluminescence reagent (SuperSignal West Pico Chemiluminescent Substrate, Cat # 34080, Thermo Fisher Scientific; 4 min, room temperature). Chemiluminescence was detected using Kodak Scientific Imaging film X-OMAT™ Blue XB (Cat # 1776699, Perkin-Elmer Life Sciences Inc., Boston, MA) processed in a Konica medical film processor (Model SRX-101A, Konica Medical Imaging Inc., Wayne, NJ). Blots exposed to Streptavidin-IRDye were washed once with TBS, twice with distilled water, and then imaged using an Odyssey Imaging System (Li-Cor).

When unconjugated 6E10 was used as the detection antibody, membranes were incubated for 1 h at room temperature in goat-anti-mouse immunoglobulin G (IgG) conjugated to HRP (Cat # 115–035-174, Jackson ImmunoResearch, West Grove, PA; 1:200,000 in TBS-T), then washed 5 X 5 min in TBS-T. These blots were developed with chemiluminescence reagent, as described above.

Blots probed with other unconjugated antibodies were incubated for 1 h at room temperature with the species-appropriate secondary antibody conjugated to an IR dye (goat-anti-mouse IgG conjugated to IRDye 800CW, 1:100,000; Cat # P/N 926–32,210; goat-anti-rabbit IgG conjugated to IRDye 680LT, 1:150,000; Cat # P/N 926–68,021; Li-Cor). Blots were then washed 5 X 5 min in TBS-T, once with TBS, twice with distilled water, and imaged using an Odyssey Imaging System (Li-Cor).

After probing with 6E10, biotin-conjugated 6E10 or Neutravidin-HRP, Western blots of brain lysates were stripped using Restore PLUS Western Blot Stripping Buffer (Cat # 46430, Thermo Scientific) for 1 h at room temperature. The blots were then washed 5 X 5 min in TBS-T, blocked with blocking buffer for 1 h at room temperature, and then incubated overnight at 4 °C with anti-α-tubulin, 5 ng/mL blocking buffer. Subsequently, membranes were incubated for 1 h at room temperature in goat-anti-mouse IgG conjugated to HRP, then washed 5 X 5 min in TBS-T. These blots were developed with chemiluminescence reagent, as described above.

## Results

### Spurious Bands in Western Blots Due to the Presence of Endogenous Biotinylated Proteins

**Fig. 1:**
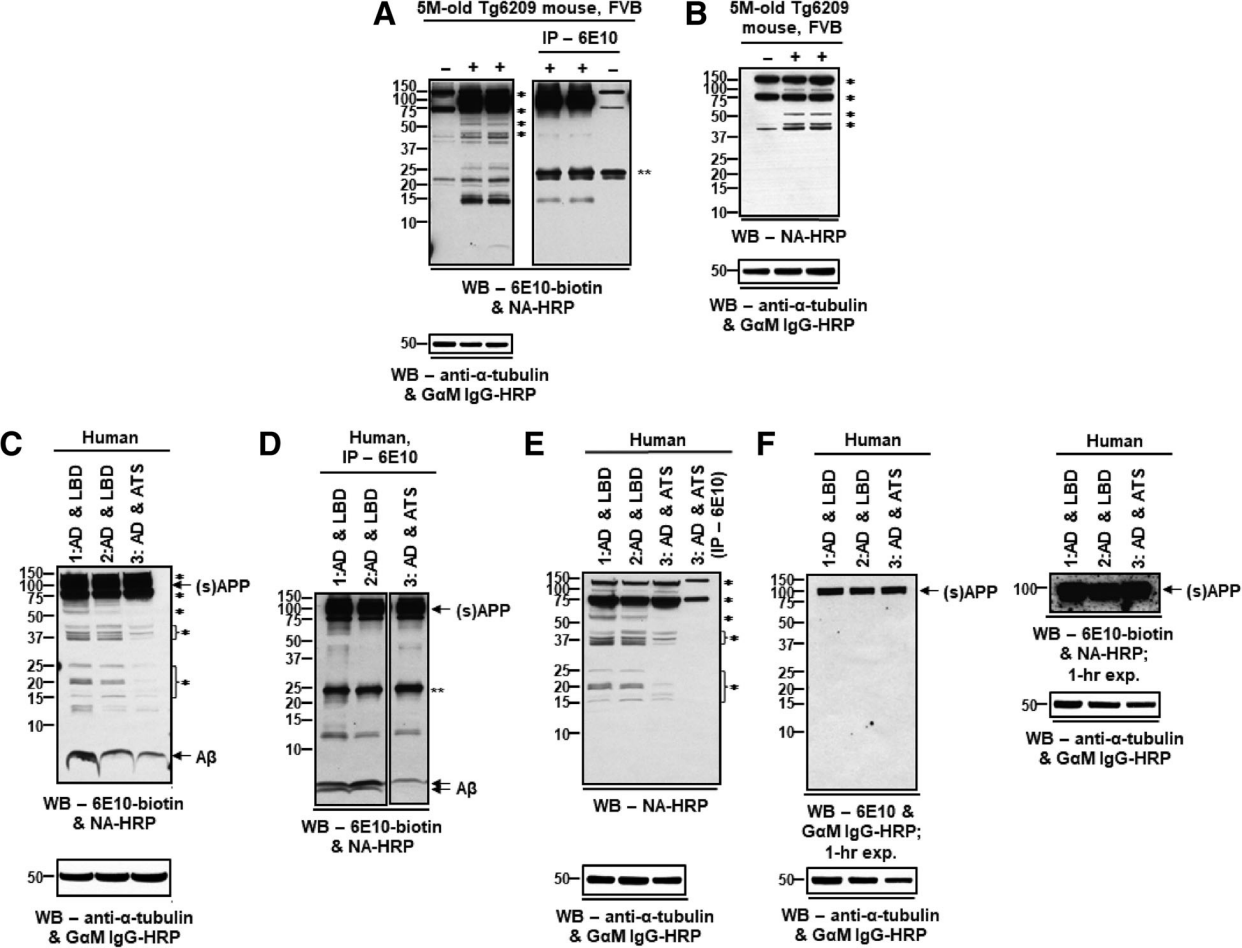
Antibody-independent signals in blots developed using Neutravidin-HRP. **a** *Left*, Western blot of mouse-brain extracts, probed with biotin-conjugated 6E10 (6E10-biotin), followed by Neutravidin-HRP (NA-HRP) and chemiluminescence reagent. *Right,* samples immunoprecipitated with 6E10 prior to blotting. Note bands marked by asterisks (*), which are eliminated (~ 46 kDa, ~ 55 kDa) or diminished (~ 75 kDa, ~ 150 kDa) when samples are immunoprecipitated prior to blotting. **, Protein G. **b** Blot of mouse-brain extracts processed with NA-HRP and chemiluminescence reagent, without exposure to antibody 6E10. Note bands marked by (*), which appear even in the absence of antibody. +, APP transgenic mice (line Tg6209); −, non-transgenic mouse. **c** Western blot of extracts from Alzheimer’s disease (AD) brains, probed with 6E10-biotin, followed by NA-HRP and chemiluminescence reagent. Lanes 1 and 2, brains had AD and Lewy body (LBD) pathology; lane 3, brain displayed AD pathology and atherosclerosis (ATS). Bands marked by asterisks (*) are also present in (**e**). (s)APP, full-length and/or α-secretase-cleaved, soluble N-terminal fragment of APP; Aβ, monomeric Aβ. **d** Brain extracts in (**c**) immunoprecipitated with 6E10; blot processed as in (**c**). Compare pattern of bands in (**c**) and (**d**). (s)APP, Aβ, and a doublet at ~ 12 kDa are immunoprecipitated. **, Protein G. **e** Blot processed with NA-HRP and chemiluminescence reagent, without exposure to antibody 6E10. IP, sample immunoprecipitated with 6E10; other lanes, samples prepared as in (**c**). Note that (s)APP, ~ 12-kDa doublet, and Aβ bands, seen when blots are exposed to anti-APP/Aβ antibody 6E10 (**c**), are absent here, and that immunoprecipitation eliminates or diminishes the non-specific bands (*). **f** *Left*, blot probed with unconjugated 6E10 followed by goat-anti-mouse IgG conjugated to HRP (GαM IgG-HRP), 1-h exposure. Only the (s)APP band is detectable. *Right*, (s)APP signal after 1-h exp. in blot probed using 6E10-biotin, followed by NA-HRP, for comparison. (Bottom panels accompanying Western blots *s*how anti-α-tubulin loading controls (see Methods))

The first artefact was revealed when we were attempting to identify amyloid precursor protein (APP) metabolites in the brains of transgenic mice that express human APP. (APP transgenic mice are used to model amyloid-related processes in AD [17, 18]). As a first step, biotin-conjugated monoclonal antibody 6E10 was used to probe Western blots of brain lysates from Tg6209 APP transgenic mice; brain lysates from age-matched, non-transgenic mice were used as negative controls. 6E10 is a commonly-used antibody that recognizes amino acids 677–681 (amino acid position in 770-amino acid isoform, Uniprot P05067; corresponds to amino acids 6–10 of Aβ) of human, but not mouse, APP [19]. The biotin-conjugated antibody was used in order to amplify any signals and increase the likelihood of detecting low-abundance APP metabolites. Bound antibody was detected by incubation of the blots with Neutravidin conjugated to HRP, followed by chemiluminescence reagent (peroxidase substrate). Three prominent bands, at approximately 22 kDa, 75 kDa and 150 kDa, were seen in samples from both transgenic and non-transgenic animals, and were immediately regarded as non-specific (in our experience, ~ 75 kDa and ~ 150 kDa bands are seen whenever an avidin derivative is used to probe blots of mouse brain lysates, regardless of the detection antibody employed). Several bands were present in transgene-positive Tg6209 mice, but absent in non-transgenic controls: a band at ~ 100 kDa (molecular weight of full-length APP or sAPPα, the soluble product formed by proteolytic cleavage of APP by α-secretase) and multiple additional bands at approximate molecular weights of 62 kDa, 55 kDa, 46 kDa, 25 kDa, and 15 kDa, which we suspected might represent SDS-stable Aβ oligomers or other APP metabolites (Fig. 1a). However, the bands at ~ 55 kDa and ~ 46 kDa were not immunoprecipitated by antibody 6E10, calling into question the identity of these bands as genuine 6E10-immunoreactive species. (Immunoprecipitation weakened, but did not eliminate, the non-specific bands at ~ 75 kDa and ~ 150 kDa, indicating that our wash conditions were not stringent enough to fully remove these highly abundant and/or “sticky” species). Indeed, the transgene-dependent ~ 46 kDa and ~ 55 kDa bands, as well as the ~ 75 kDa and ~ 150 kDa bands, were present when blots were exposed only to Neutravidin-HRP followed by chemiluminescence reagent (Fig. 1b), indicating that these bands represented species that reacted either with the Neutravidin (i.e., endogenous biotinylated proteins) or with the chemiluminescence reagent (i.e., endogenous peroxidases whose activity survived detergent extraction and SDS-PAGE). We concluded that the ~ 46 kDa and ~ 55 kDa bands seen in Western blots of Tg6209 lysates were *transgene-dependent*, *antibody-independent* artefacts caused by the detection protocol. Had we only performed Western blots using a sample control, we could have erroneously concluded that these bands represented actual APP metabolites. The failure to immunoprecipitate these bands aroused suspicion, but in the absence of further experiments, might have been attributed to epitope masking under native conditions.

Similar artefacts were also found to be present in human samples. In blots of lysates from AD patients, we observed bands at ~ 4.5 kDa (the molecular weight of Aβ monomers), ~ 100 kDa, and multiple additional, distinct bands at approximately 62 kDa, 55 kDa, 37–45 kDa, and 12–25 kDa (Fig. 1c). The non-specific bands at ~ 75 kDa and ~ 150 kDa were also observed in blots of human samples. As was the case for the mouse samples, several of these bands were not immunoprecipitated by 6E10 (i.e., bands ~ 55 kDa, 37–45 kDa, and 15–25 kDa) (Fig. 1d), and these same bands persisted when biotin-conjugated 6E10 was omitted from the Western blotting protocol (Fig. 1e), indicating that these bands represented species that reacted either with the Neutravidin or with the chemiluminescence reagent. Conversely, the ~ 4.5 kDa band, a doublet of bands at ~ 12 kDa, and the heavy band at ~ 100 kDa were immunoprecipitated by 6E10 and were absent from blots exposed only to Neutravidin-HRP and chemiluminescence reagent, supporting their identity as genuine 6E10-immunoreactive species (Fig. 1d and e). (The ~ 62-kDa band was difficult to distinguish after immunoprecipitation–it appeared to be present, but was largely obscured in “smears” in the region of ~ 55–75 kDa). When blots were exposed to chemiluminescence reagent without Neutravidin-HRP (i.e., blots were probed using unconjugated 6E10 followed by goat-anti-mouse IgG-HRP secondary antibody; Fig. 1f), the bands seen in Fig. 1e failed to appear, strongly suggesting that these bands represent endogenous biotinylated molecules. Unfortunately, without the signal amplification provided by the biotin-avidin detection system, the ~ 4.5-kDa (likely monomeric Aβ) and ~ 12-kDa bands were not detectable in blots probed using 6E10/secondary antibody-HRP even at an exposure time (i.e., 1 h) when the signal intensity was maximal using this detection system. In order to avoid the detection of endogenous biotinylated proteins, while taking advantage of the amplification afforded by a biotin-avidin detection system, we recommend that target proteins be immunoprecipitated prior to Western blotting (as shown in Fig. 1a (*right)* and 1d)**.**

### Introduction of Exogenous Proteins that Non-specifically Bind Detection Antibodies

As part of our standard protocol for the preparation of brain lysates, we deplete the lysates of endogenous immunoglobulins using either Protein A or Protein G conjugated to Sepharose beads. This depletion step serves to eliminate mouse immunoglobulins that: i) would be detected when anti-mouse secondary antibodies are used in subsequent immunoblotting experiments, and ii) might bind to Protein A or Protein G in immunoprecipitation experiments. Surprisingly, this depletion step led to a conspicuous and potentially very misleading artefact when we attempted to immunopurify Aβ species from brain lysates.

**Fig. 2:**
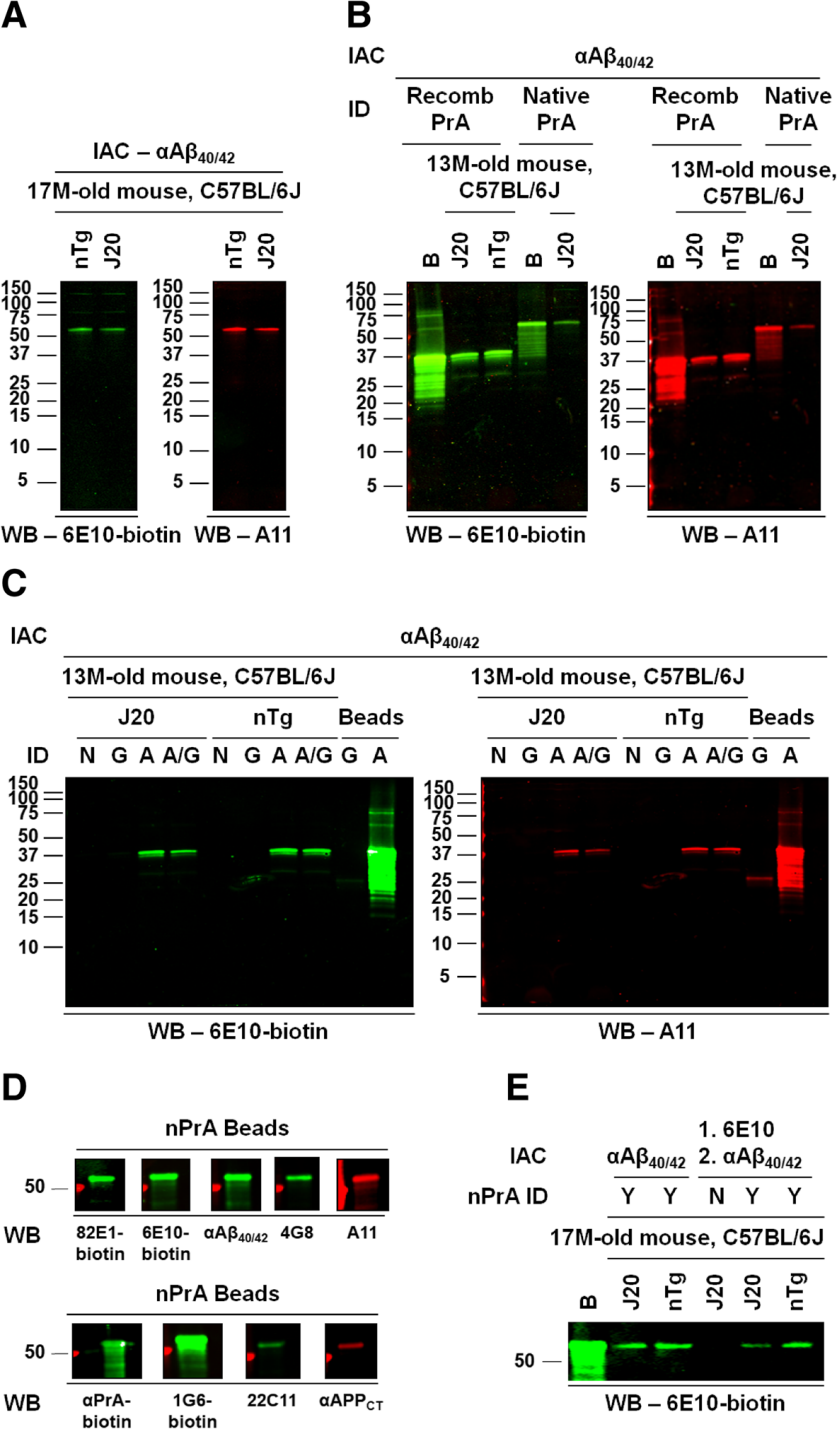
Protein A contamination leads to artefactual bands during immunoaffinity purification. **a** Similar bands are detected by Western blot after immunoaffinity purification (anti-Aβ_40/42_ immunoaffinity matrix) of brain lysates from hAPP-J20 (J20) or non-transgenic (nTg) mice. *Left*, blot probed with 6E10-biotin, followed by IR-conjugated Neutravidin; *right*, blot probed with A11 followed by IR-conjugated goat-anti-rabbit IgG. Because monoclonal antibody 6E10 does not recognize mouse Aβ/APP, bands cannot represent genuine 6E10 immunoreactivity. **b** Molecular weights of bands detected by Western blot depend upon the source of Protein A used for immunodepletion. Brain lysates from J20 or nTg mice were depleted of endogenous immunoglobulins using either recombinant (recomb) or native Protein A (PrA), conjugated to Sepharose beads, and then subjected to anti-Aβ_40/42_ immunoaffinity purification. Eluates were analyzed in Western blots probed with 6E10 (*left*, green) or A11 (*right*, red). “B” marks material recovered after boiling of Protein A-conjugated Sepharose beads. **c** Bands present only in samples exposed to Protein A. Brain lysates from J20 or nTg mice underwent anti-Aβ_40/42_ immunoaffinity purification, either without prior depletion of endogenous immunoglobulins (N) or following depletion using Protein A (recombinant) and/or Protein G. Western blots of the eluates from the anti-Aβ_40/42_ matrix, probed with 6E10 (*left*, green) or A11 (*right*, red). “Beads” marks material recovered after boiling of Protein A- or Protein G- conjugated Sepharose beads. **d** Multiple antibodies react with denatured Protein A. Protein A-conjugated Sepharose beads were boiled in SDS-PAGE loading buffer, and recovered material was subjected to SDS-PAGE and Western blot. Blots were probed using a panel of antibodies (Table 1). **e** Brain lysates from J20 or nTg mice were subjected to single-step (anti-Aβ_40/42_) or sequential (6E10 followed by anti-Aβ_40/42_) immunoaffinity purification. IAC, immunoaffinity capture. nPrA ID, lysate immunodepleted using (native) Protein A-Sepharose prior to immunoaffinity purification; Y, yes; N, no. Blots probed with 6E10-biotin, followed by IR-conjugated Neutravidin. “B,” material recovered after boiling Protein A-Sepharose

When brain lysates from hAPP-J20 transgenic mice were subjected to immunoaffinity purification using anti-Aβ_40/42_-conjugated magnetic beads, and the eluate from the beads was then analyzed by Western blot, a single ~ 55-kDa band was revealed in blots probed using either 6E10 or the “oligomer-specific” antibody A11 [20]. Our initial interpretation of this finding was that this band represented an isolated Aβ oligomer or an SDS-stable protein complex that contained Aβ. However, similar bands were detected by 6E10 and A11 when brain lysates from non-transgenic mice were subjected to the same immunoaffinity-purification protocol (Fig. 2a). As 6E10 is human-specific, the band revealed by this antibody after immunopurification of lysates from non-transgenic animals cannot represent an authentic APP metabolite.

We suspected that the ~ 55-kDa band might represent Protein A contamination, due to incidental observations in earlier, unrelated experiments where a similarly migrating band was seen in various blots from lysates depleted using native protein A (as used for the lysates in the immunopurification experiments illustrated in Fig. 2a) but not recombinant Protein A or Protein G. We therefore prepared a new series of brain lysates, immunodepleted with native Protein A (nProtA), recombinant Protein A (rProtA), and/or Protein G. When subjected to the immunopurification protocol followed by Western blot analysis using 6E10 or A11, the lysates depleted with rProtA yielded an ~ 37-kDa band, rather than the ~ 55-kDa band seen after “purification” of lysates depleted using nProtA (Fig. 2b). No 6E10- or A11-binding species were seen after purification of lysates depleted with only Protein G (Fig. 2c).

Multiple antibodies (Table 1) that we use in our studies of APP and its metabolites were found to bind to denatured Protein A (Fig. 2d).

Finally, we subjected brain lysates to a sequential immunopurification protocol that has been reported to yield Aβ oligomers [21]. Lysate was first applied to 6E10-conjugated magnetic beads, then the eluate from these beads was applied to anti-Aβ_40/42_-conjugated beads. Probing with 6E10, Western blots of the eluate from these beads showed a single, prominent band at ~ 55 kDa when using lysates that had been immunodepleted using (native) Protein A-Sepharose beads prior to immunoaffinity purification. This band was observed in lysates from both transgenic and non-transgenic animals but was absent in lysates that had not been exposed to Protein A (Fig. 2e).

**Fig. 3:**
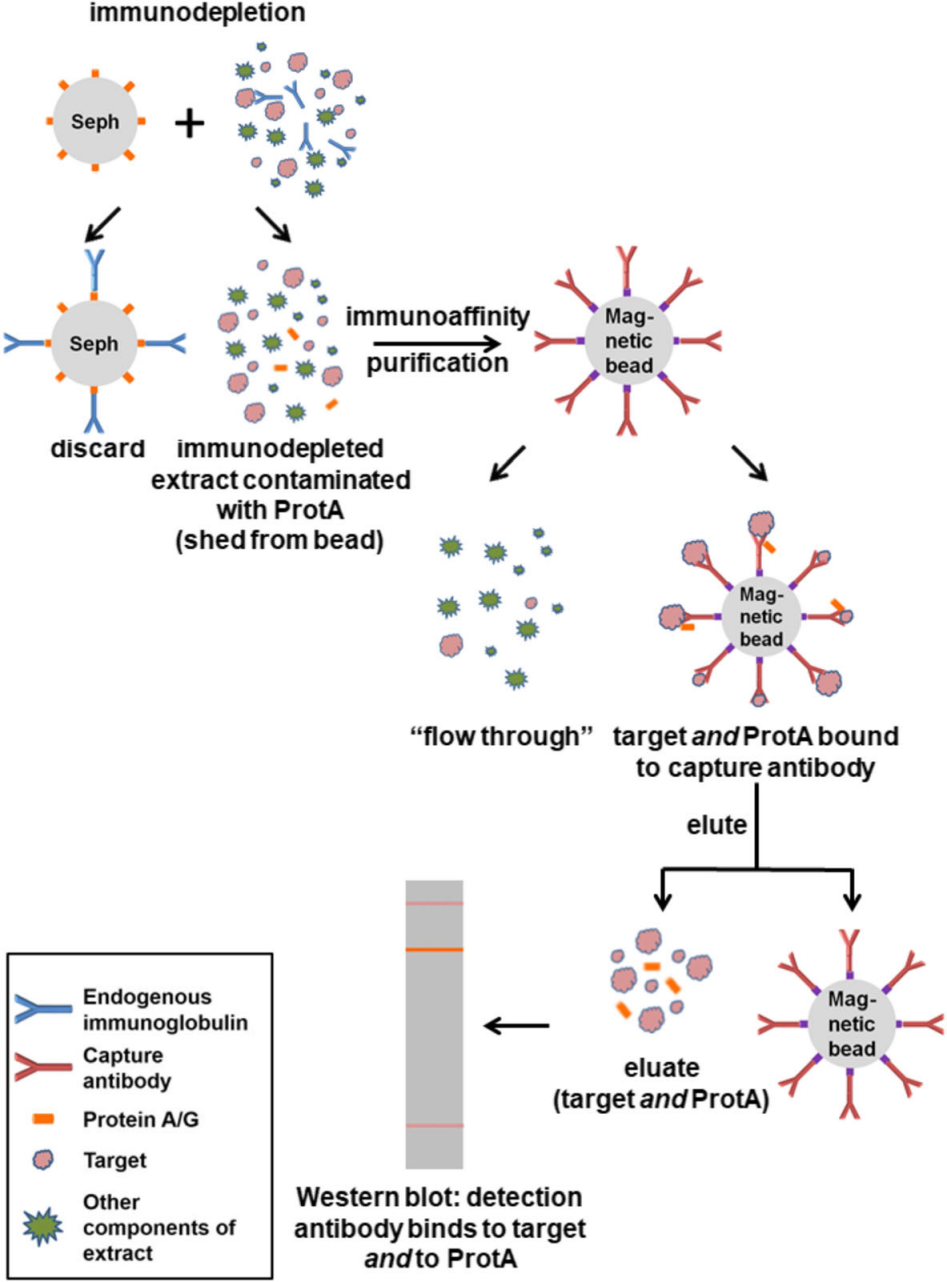
Diagram illustrating genesis of Protein A artefact in immunoaffinity purification procedure. Brain lysates were depleted of endogenous immunoglobulins using Protein A-conjugated Sepharose beads. During this step, some Protein A shed from the beads and contaminated the brain lysate. Upon exposure to the immunoaffinity matrix, some of the Protein A bound to the Fab region of the immobilized antibodies. This Protein A was then eluted from the matrix by the low pH elution buffer used to recover the target species. During subsequent Western blotting of the eluate from the antibody-conjugated magnetic beads, the denatured Protein A was still able to bind detection antibody

We conclude that the 6E10- and A11-positive bands represent binding of the antibodies to Protein A that shed from the Sepharose beads during the depletion step and carried through the immunoaffinity purification steps (Fig. 3). We were initially surprised that Protein A would be bound by, and then eluted from, the antibody-conjugated beads; however, in addition to binding the Fc region, Protein A has been shown to bind the Fab region of some mouse monoclonal antibodies, and this binding is disrupted at low pH, such as that used for elution in our protocol [22, 23].

## Discussion

Critical to the correct interpretation of Western blots is the ability to determine which bands represent the target proteins and which are artefacts. Identification of the correct bands can be especially challenging when the molecular weights of the target species are uncertain, which is often the case when studying abnormal, disease-associated protein aggregation or cleavage. We here describe two antibody-independent artefacts that may cloud the interpretation of immunoblotting experiments: i) the detection of endogenous biotinylated proteins when avidin-biotin systems are used for antibody detection, and ii) the introduction of exogenous proteins–Protein A, in the example illustrated here–that bind detection antibodies.

The potential interference of endogenous biotin in immunohistochemistry [24, 25] and immunoblotting [11–13] has been recognized for decades. Indeed, the direct binding of labeled avidin derivatives to protein blots is used to study endogenous biotinylated proteins [26, 27]. The brain has been reported to be one of the tissues with relatively high levels of endogenous biotin [24], so perhaps it should not be surprising that we found spurious bands due to endogenous biotin in our Western blots. But what was surprising–and what initially led us to believe that these bands represented actual 6E10-immunoreactive APP/Aβ–was the dramatic difference between Tg6209 mice, which overexpress wild-type human APP, and non-transgenic animals in the expression of several of these bands. However, it should be noted that the APP-transgene-dependent expression of biotinylated proteins seen in brain lysates from Tg6209 mice was not seen in Tg2576 mice [28], which overexpress human APP with the AD-linked “Swedish” mutation. In addition to carrying different APP transgenes, the two lines are maintained on different genetic backgrounds–FVB for Tg6209 and B6SJL for Tg2576.

We did not seek to identify the molecular species giving rise to the antibody-independent, Neutravidin-dependent bands in our blots, although it should be noted that carboxylases are probably the best studied biotinylated proteins, and the prominent bands at ~ 75 kDa and ~ 125–150 kDa in both transgenic and non-transgenic mice and in humans might represent β-methylcrotonyl-CoA carboxylase (72 kDa) and/or propionyl-CoA-carboxylase (74 kDa) and pyruvate carboxylase (128 kDa) [27].

We also show that Protein A–shed from beads added to deplete lysates of endogenous immunoglobulins–bound to, and was eluted from, immunoaffinity matrices designed to capture APP and/or Aβ. This Protein A then bound detection antibodies when the immunoaffinity eluates were analyzed by Western blot. Although Protein G has also been reported to bind to Fab [22, 29], under our experimental conditions, Protein G did not cause a problem similar to that which we encountered when using Protein A. We do not know why we found these differences, but we speculate that there may have been less shedding of Protein G from the Sepharose beads or that the Protein G did not have as strong an affinity as Protein A for the antibodies used in our immunoaffinity purifications. At this time, we recommend that a decision as to whether to use Protein A or Protein G in any particular experiment be made empirically.

Much attention has been paid to the potential for poorly-characterized antibodies to lead to misleading results [9, 10, 30]. However, as shown here, *antibody-independent* artefacts may also occur when antibodies are used to detect or isolate other proteins. One way to monitor such artefacts is to include an “irrelevant” antibody as an experimental control (e.g., running a parallel protocol in which the antibody directed against the target of interest is replaced by a non-specific antibody). In the past, when laboratories generated their own polyclonal antibodies, it was standard practice to include pre-immune serum as a control. With the current dependence on commercial antibodies, it may not be possible to obtain pre-immune serum from the animal(s) that generated the “specific” antibodies, but normal sera or immunoglobulins from the same species should be readily available. When using monoclonal antibodies–even those with validated specificity–controls should include a non-specific immunoglobulin, preferably of the same isotype. As our studies illustrate, it is not sufficient to include as controls samples from animals known to lack the target protein, since bands attributable to endogenous biotinylated proteins can be seen in brain lysates from transgenic animals but not from non-transgenic mice (as seen in our experiments using the Tg6209 line of APP transgenic mice). Although the use of an “irrelevant antibody” control will not reveal the exact source of the artefact, this strategy is, to the authors’ knowledge, the most efficient way to detect artefactual differences between experimental and control groups.

## Additional file

Additional file 1: **Figure S1.** Survey of methodological controls in immunocapture and immunoblotting studies. (PDF 143 kb)

## Abbreviations

1,10-PTH: 1,10-phenanthroline monohydrate
AD: Alzheimer’s disease
APP: Amyloid precursor protein
Aβ: Amyloid-β
BCA: Bicinchoninic acid
EDTA: Ethylenediaminetetraacetic acid
Fab: Antigen-binding fragments
Fc: Crystallizable fragments
hAPP-J20: Transgenic mouse line B6.Cg-Zbtb20^Tg(PDGFB-APPSwInd)20Lms^/2Mmjax, which overexpresses human amyloid precursor protein with the *Swedish* and *Indiana* mutations linked to familial Alzheimer’s disease
HRP: Horseradish peroxidase
IAC: Immunoaffinity capture
IgG: Immunoglobulin G
IP: Immunoprecipitation
NP-40: Nonidet P-40
nProtA: Native Protein A
PAGE: Polyacrylamide gel electrophoresis
PMSF: Phenylmethylsulfonyl fluoride
recomb: Recombinant
rProtA: Recombinant Protein A
sAPPα: The soluble product formed by proteolytic cleavage of APP by α-secretase
SDS: Sodium dodecyl sulfate
TBS: Tris-buffered saline
TBS-T: Tris-buffered saline with 0.1% (v/v) Tween 20
TEA: Triethanolamine
Tg2576: Transgenic mouse line B6;SJL-Tg(APPSWE)2576Kha, which overexpresses the human amyloid precursor protein with the *Swedish* mutation linked to familial Alzheimer’s disease
Tg6209: Transgenic mouse line Tg(HuAPP_695_.WTmyc)6209, which overexpresses wild-type human amyloid precursor protein, carboxy-terminally tagged with a 12-amino-acid-long c-myc protein fragment
Tris–HCl: Tris(hydroxymethyl)aminomethane-hydrochloric acid
Triton X-100: Polyethylene glycol p-(1,1,3,3-tetramethylbutyl)-phenyl ether
v/v: Volume/volume
w/v: Weight/volume
WB: Western blotting
