# Supplementary material for: A Cautionary Tale: Endogenous Biotinylated Proteins and Exogenously-Introduced Protein A Cause Antibody-Independent Artefacts in Western Blot Studies of Brain-Derived Proteins
Source: Biol Proced Online. 2019 Apr 18;21:6. doi: 10.1186/s12575-019-0095-z (PMC6474067; doi:10.1186/s12575-019-0095-z)
Supplement: Supplementary file 1 — Figure S1. Survey of methodological controls in immunocapture and immunoblotting studies. (PDF 143 kb) [file 12575_2019_95_MOESM1_ESM.pdf]

Supplementary Figure 1. Survey of methodological controls in immunocapture and immunoblotting studies. Studies employing immunoblotting , immunoprecipitation, and immunoaffinity purification—techniques most directly analogous to the methods used in the current paper—were surveyed. Four searches were conducted in PubMed: 1) search designed to capture such studies in the field of Alzheimer's research conducted during the preceding year; 2) search designed to capture studies related to β-amyloid oligomers, the specific subject of our own research that led to the identification of the artefacts described in the current paper; 3) more focused search to find papers that may have used a biotin amplification system when immunologically characterizing amyloid peptides; and 4) search designed to find any papers that employed a biotin amplification system in immunoblotting studies more generally, conducted during the preceding year. These searches yielded a total of 751 studies to which we had access to the full text. Of these, 230 were excluded—primarily because they did not include an appropriate immunoblotting or immunocapture technique. Of the remaining 521 studies, only 56 (< 11 percent) mentioned a methodological control for the immunoblotting or immunocapture experiment (as opposed to a control for experimental condition—e.g., disease vs. healthy control, drug treated vs. vehicle-treated animal, cells transfected with coding DNA vs empty vector). Search dates are shown in parentheses, and the number of papers returned by each search is shown in bold. Detection methods for immunoblots are shown: HRP-conjugated secondary antibody, HRP-conjugated secondary antibody followed by chemiluminescent reagent; Unspecified ECL, chemiluminescent reagent (peroxidase substrate) with unspecified HRP-conjugated detection reagent; Biotin … HRP, biotin-avidin-HRP amplification followed by chemiluminescent reagent; Infrared, detection reagent conjugated to infrared dye; Other, alkaline phosphatase- or fluorophore-conjugated detection reagents; Unknown, citations provided but detailed methods not provided within paper. ECL, enhanced chemiluminescence; IP, immunoprecipitation or immunoaffinity purification; ICC, immunocytochemistry; IHC, immunohistochemistry.

Search 1 (2018-12-29): **414**
Search (((immunoblot OR western blot OR immunopurification OR immunoaffinity OR immunoprecipitation)) AND Alzheimer's) AND (Publication Date: 2017/12/29 - 2018/12/29)

Search 2 (2019-01-12): **191**
Search (((immunoblot OR western blot OR immunopurification OR immunoaffinity OR immunoprecipitation)) AND amyloid) AND (oligomer OR aggregate)

Search 3 (2019-01-04): **87**
Search (((immunoblot OR western blot OR immunopurification OR immunoaffinity OR immunoprecipitation)) AND *biotin*) AND amyloid

Search 4 (2019-01-03): **120**
Search ((((immunoblot OR western blot OR immunopurification OR immunoaffinity OR immunoprecipitation)) AND *biotin*)) AND (Publication Date: 2018/01/03 - 2019/01/03)

↓

Total returned by searches: 812
Unique: 799
English: 785
Access to full-text: 751

→

Excluded: 230

Reviews (6), dUTP-biotin nick-end labeling (TUNEL) (30), surface biotinylation (49), protocols/methods (15), chromatin immunoprecipitation (5), biotin pull-down analysis (biotinylated RNA) (16) , biotin tracer to test barrier function (4), biotin switch to detect S-nitrosylation, S-sulfhydration (8), biotinylated compounds (non-RNA; includes receptor ligands, drugs, peptides) to use as bait (16), , IHC, ICC (7), publisher's correction (1), unclear why returned by search (no blots, IPs) (3) , biotinylated dextran for cell tracing (1), biotin as molecular tag for capture or detection of synthetic peptides (11) , proximity biotinylation/ligation (3), in vivo biotinylation (2), biotin to link (via avidin) to other molecule/complex (1), blots of synthetic targets (screening new antibodies) (2), studies of endogenous biotinylation (biotinylated proteins, synthetic enzymes) (4), acyl-biotin exchange assay (palmitoylation) (2), non-Western blot (non-antibody detection) (6), "in-cell Western blot" (1), biotinylated tumor-targeting agents (2), blots of synthetic targets (protein aggregation, etc.) (4), biotinylated peptide for ECL assay on bead (1), click chemistry with biotin-azide (1), αLISA (1), generation of biotin-containing probe (1), proteomic study -immunoaffinity depletion of highly abundant proteins prior to mass spec (1), synthetic peptides, in vitro (cell-free): 26

↓

521 eligible studies

<u>Western blot</u>	<u>Dot blot</u>
HRP-conjugated secondary antibody: 308	HRP-conjugated secondary antibody: 10
Unspecified ECL: 106	Unspecified ECL: 7
Biotin … HRP: 4	Biotin … HRP: 1
Infrared: 27	Infrared: 2
Other: 7	Other: 1
Not stated: 6	Not stated: 3
Unknown: 11 (authors provide citations)	

<u>Immunopreciptiation/immunopurification</u>: 81

↓

Methodological control: 56

<u>Immnoblotting</u>	<u>Immunopreciptiation/immunopurification*</u>
Irrelevant antibody as primary antibody: 3	Non-specific or irrelevant immunoglobulin as capture antibody: 24
No primary antibody: 7	Immunodepletion of target species prior to immunocapture: 3
Sample from knock-out mouse: 4	Protein A/G beads (no antibody) + sample: 4
Preabsorbtion of antibody with antigen: 6	No sample: 2
Non-target peptide: 2	Protein A/G beads only (no antibody or sample): 3

*An additional 3 immunoprecipitation studies included a preclearing step in which sample was incubated with Protein A/G beads prior to incubation with capture antibodies/beads, to remove endogenous immunoglobulins and any other species that might non-specifically stick to the beads. These studies did not contribute to the "methodological controls total."
